# Who is a ‘healthy subject’?—consensus results on pivotal eligibility criteria for clinical trials

**DOI:** 10.1007/s00228-016-2189-8

**Published:** 2017-01-07

**Authors:** Kerstin Breithaupt-Groegler, Christoph Coch, Martin Coenen, Frank Donath, Katharina Erb-Zohar, Klaus Francke, Karin Goehler, Mario Iovino, Klaus Peter Kammerer, Gerd Mikus, Jens Rengelshausen, Hildegard Sourgens, Reinhard Schinzel, Thomas Sudhop, Georg Wensing

**Affiliations:** 1 -kbr- clinical pharmacology services, D-60431 Frankfurt am Main, Germany; 2 Institute of Clinical Chemistry and Clinical Pharmacology, University of Bonn, Sigmund-Freud-Str. 25, 53127 Bonn, Germany; 3 SocraTec Research and Development GmbH, D-99084 Erfurt, Germany; 4 clinphase®, D-63454 Hanau, Germany; 5 National Association of Statutory Health Insurance Funds, Medicinal Product Department, 10117 Berlin, Germany; 6 Gruenenthal GmbH, Gruenenthal Innovation—Development—Clinical Development—Clinical Pharmacology, D-52099 Aachen, Germany; 7 Boehringer Ingelheim Pharma GmbH & Co. KG, Translational Medicine and Clinical Pharmacology, D-88397 Biberach/Riss, Germany; 8 Department of Clinical Pharmacology and Pharmacoepidemiology, University of Heidelberg, D-69120 Heidelberg, Germany; 9 Gruenenthal GmbH, Gruenenthal Innovation—Research—Translational Science & Strategy—Early Clinical Science, D-52078 Aachen, Germany; 10 Sourgens Consulting, D-80797 Munich, Germany; 11 vasopharm GmbH, D-97076 Würzburg, Germany; 12 Federal Institute for Drugs and Medical Devices (BfArM), D-53175 Bonn, Germany; 13 Bayer Pharma AG, Pharmaceutical Division Clinical Pharmacology Cardiovascular/Hematology (Primary Care), D-42096 Wuppertal, Germany

**Keywords:** Healthy subject, Phase I, Safety parameters, Inclusion/exclusion criteria, Stopping rules, Investigational medicinal product

## Abstract

**Introduction/Methods:**

A discussion forum was hosted by the German not-for-profit Association for Applied Human Pharmacology (AGAH e.V.) to critically review key eligibility criteria and stopping rules for clinical trials with healthy subjects, enrolling stakeholders from the pharmaceutical industry, contract research organisations, academia, ethics committees and competent authority.

**Results:**

Pivotal eligibility criteria were defined for trials with new investigational medicinal products (IMPs) or with clinically established IMPs. In general, a pulse rate ranging between 50 and 90 beats/min is recommended for first-in-human (FIH) trials, while wider ranges seem acceptable for trials with clinically established IMPs, provided there are no indications of thyroid dysfunction. Hepatic laboratory parameters not to exceed the upper limit of normal (ULN) comprise ALT (alanine aminotransferase) and AST (aspartate aminotransferase) in FIH trials, whereas slight elevations (10% above ULN) seem acceptable in trials with clinically established IMPs without known hepatotoxicity. A normal renal function is required for any clinical trial in healthy subjects. A risk-adapted approach for stopping rules was adopted. Stopping rules for an individual subject are one adverse event of severe intensity or one serious adverse event. In case of a severe adverse event, some stakeholders demand a causal relationship with the IMP (i.e. an adverse reaction). Stopping rules for a cohort are one serious adverse reaction or ≥50% of subjects experiencing any adverse reaction of moderate or severe intensity.

**Consequences:**

The application of this consensus resulted in a reduction in protocol deficiencies issued by the competent authority.

## Introduction

Subsequent to the most recent tragedy that happened in a phase I trial with a new compound under investigation, safety measures for trials in healthy volunteers are in public focus [1, 2]. Key safety parameters relate to inclusion and exclusion criteria defining a healthy population as well as to stopping rules for further exposure to the investigational medicinal product (IMP).

Different definitions of ‘health’ and ‘healthy volunteer’ exist: the WHO defines ‘health’ as ‘a state of complete physical, mental and social well-being and not merely the absence of disease or infirmity’ [3]. The Royal College of Physicians defines a ‘healthy volunteer’ as ‘an individual who is not known to suffer any significant illness relevant to the proposed study, who should be within the ordinary range of body measurements’ [4] whereas ‘The Textbook of Pharmaceutical Medicine’ refers to ‘an individual who is in good general health, not having any mental or physical disorder requiring regular or frequent medication’ [5]. However, these definitions do not provide an answer how ‘health’ can be ascertained before and during the course of a clinical trial in healthy subjects.

## Purpose

There are no guidelines in place defining acceptable normal ranges for key safety parameters permitting enrolment of a healthy subject into a phase I clinical trial. Moreover, timing and frequency of safety assessments are also a matter of debate. As a consequence, questions on inclusion and exclusion criteria as well as stopping rules are raised by competent authorities during approval of phase I protocols and may cause uncertainty for the sponsor and delay of clinical trial authorisations.

To overcome respective deficiencies in clinical trial protocols identified by the German competent authority, three informal discussion forums were organised by the German Association for Applied Human Pharmacology (Arbeitsgemeinschaft für angewandte Humanpharmakologie, AGAH e.V.). The AGAH is a scientific medical not-for-profit organisation dedicated to facilitate research activities and to provide training in explorative drug development and human pharmacology.

## Methods

Stakeholders involved in clinical medicines development originating from the pharmaceutical industry, contract research organisations, competent authorities, ethics committees and academia in Germany worked on a consensus process. The process included a review of the available literature and the discussion of knowledge and exchange of experiences and opinions of the stakeholders. Discussion focused on normal ranges for cardiovascular parameters and key safety laboratory parameters (liver and kidney) as well as stopping rules in relation to the risk classification of the investigational medicinal products (IMP). Consensus was reached after lively debate and review of meeting minutes if no objection was raised by the stakeholders involved.

## Results

### General aspects

Phase I trials in healthy subjects comprise different types of trials without therapeutic intent with (1) clinically not established substances (e.g. first-in-human trials; FIH trials) as well as (2) clinically established medicines that are regarded as rather safe (e.g. bioequivalence trials for generic medicines applications). The latter will be referred to as ‘*trials with clinically established IMPs*’.

The choice of safety parameters in FIH trials needs to consider the target organs of toxicity determined in nonclinical testing. Care should be taken if nonclinical testing has not shown relevant toxicities. A lack of toxicological findings does not implicate that an IMP is ‘safe’. Therefore, toxicology cannot guide the investigator. Such IMPs pose a very high risk factor by themselves; a scientific advice with the competent authority is recommended.

Trials with healthy subjects require risk minimisation to the lowest possible level. At all times, the investigator is in charge of medical surveillance and clinical care for the subject. Defining threshold values for inclusion in a trial serves to ascertain the health status of a trial subject. Safety parameters outside normal ranges are *not always and not* per se clinically relevant. Changes within the normal range, on the other hand, might be indicative of noteworthy findings. The interpretation of safety parameters has to take into account effects caused by the drug, by intermittent diseases (e.g. a common cold) as well as by the highly standardised conditions during the trial (e.g. deprivation of nicotine and caffeine, lack of exercise).

Thresholds have methodological implications. ‘Postdosing’ values—indicative of potential drug effects—need to be discernable from ‘predosing’ values. This requires a recognisable signal to noise ratio.

### Timing of screening examinations

The health status of a subject in a clinical trial has to be assessed (1) during the screening period to determine eligibility and (2) during the course of the trial to determine maintenance of eligibility and potential adverse drug effects.

Screening assessments are usually based on medical history, physical examination, safety laboratory, vital signs, ECG, and check of inclusion and exclusion criteria. Time windows differ between FIH and trials with ‘clinically established IMPs’ and should be adopted as indicated in Table 1.

**Table. 1:** Time windows for screening examinations

First-in-human trials	•Check laboratory values and inclusion/exclusion criteria within 3 days prior to first dosing
	•If screening performed earlier than 3 days prior to first dosing, repeat laboratory assessments and check whether relevant changes/important events occurred
Trials with ‘clinically established IMPs’ (e.g. bioequivalence trials for generic medicines applications)	•Screening examination usually between −21 and −1 days prior to first dosing; take risk-adapted approach

*IMP* investigational medicinal product

The decision whether inclusion/exclusion criteria are met/not met should be made at the screening examination. Baseline evaluations immediately before dosing are not intended to reassess inclusion and exclusion criteria but to serve as reference values for the trial interventions. Thus, assessments at baseline and at the final visit are indispensable for the scientific interpretation of the trial results as well as to document the health status of a trial subject. Notably, stopping rules defined in the trial protocol also apply to baseline assessments.

Frequency and extent of safety monitoring measures should be defined for the individual trial on a case-by-case decision, considering the pharmacological characteristics of the substance (pharmacodynamics, pharmacokinetics, safety profile) and the nature of the trial.

### Safety parameters identified as frequent issues by the German competent authority ‘BfArM’

#### Heart/pulse rate

##### Method of choice

The procedure to measure heart/pulse rate (e.g. method of assessment, body position and length of resting period) should be outlined in the trial protocol. Heart/pulse rate should be assessed over a period of 60 s to avoid error extension. If shorter recordings as e.g. from automatic outputs like ECG strips taken over 25 s or readings from blood pressure machines indicate values outside of the intended reference ranges, reassessments over 60 s are recommended.

##### First-in-human trials

A resting heart rate between 50 and 90 beats per minute (bpm) is recommended as inclusion criterion. Subjects with heart rate values between 45 and 50 bpm may be enrolled in case they have a normal thyroid function, no clinical symptoms associated with the bradycardia and no apparent signs of other diseases causing bradycardia (e.g. hypothyroidism). This can require additional medical examinations of the subject on a risk-based approach (see Table 2). Subjects with heart rate <45 bpm should not be enrolled in FIH trials.

**Table. 2:** Heart/pulse rate—normal ranges/clinically acceptable ranges

First-in-human trials	•Range between 50 and 90 bpm is recommended
	• Some stakeholders consider heart/pulse rate <50 and ≥45 bpm acceptable in case of normal thyroid function (medical history, physical examination, normal TSH) and no signs of diseases associated with bradycardia plus, if required, normal cardiological examination (including echocardiography and ergometric stress test); take risk-adapted approach
Trials with ‘clinically established IMPs’ (e.g. bioequivalence trials for generic medicines applications)	• Range between 50 and 90 bpm is recommended • Potentially, heart/pulse rate <50 and ≥45 bpm acceptable in case of normal thyroid function (medical history, physical examination, TSH) and no signs of diseases associated with bradycardia (e.g. orthostasis and dizziness) •Some stakeholders consider heart/pulse rate <45 bpm acceptable in case of above stated criteria plus normal cardiological examination (including echocardiography and ergometric stress test); take risk-adapted approach

*IMP* investigational medicinal product, *TSH* thyroid-stimulating hormone

##### Trials with ‘clinically established IMPs’

For heart/pulse rates at screening compliant with the categories 50 to 90 bpm and <50 and ≥45 bpm, refer to Table 2. Some stakeholders see values below 45 bpm as an option; this requires normal cardiac function confirmed via echocardiography and stress test.

The decision to enrol subjects with a heart/pulse rate outside the range of 50 to 90 bpm at screening needs to be justified in the trial protocol on a risk-based approach.

### ECG

The QT interval corrected by the Fridericia formula (QTcF) should be within normal ranges as defined in the clinical trial protocol. First-degree atrioventricular (AV) block seems acceptable if heart/pulse rate complies with the inclusion criteria (see Table 2) and the AV block is not interpreted as a sign of cardiac dysfunction/disease. This needs to be defined in the trial protocol. Current guidance documents have to be respected. Alterations of the baseline ECG should not obscure potential drug effects.

### Blood pressure

In general, threshold values concerning blood pressure in phase I trial protocols do not seem to be a frequent matter of debate. Acceptable ranges for enrolment should be defined in the protocol. To get reliable and reproducible results, it is recommended to take measurements at screening after 5 min sitting at rest in an upright position with feet flat on the floor and to use a cuff in the correct size.

### Laboratory parameters

#### First-in-human trials

Liver as well as kidney parameters should routinely be checked and not exceed the upper normal limit (ULN). Amylase and lipase, at least partially reflecting pancreatic function depending on which isoforms are assessed, should be interpreted with caution and in clinical context. In case of Gilbert’s Syndrome, bilirubin values should be interpreted in clinical context as even elevations ≥50% above ULN do not necessarily reflect clinically relevant alterations (see Table 3). In certain cases, the deficiency in bilirubin transport might reduce the clearance of a drug substance, alter the PK results of the trial and pose a potential risk to the subject.

**Table. 3:** Laboratory parameters—normal ranges/clinically acceptable ranges

First-in-human trials	•Relevant hepatic parameter not to exceed ULN: ALT, AST, bilirubin (except in case of Gilbert’s disease*) *In case of Gilbert’s disease, elevated bilirubin not clinically relevant, yet may hamper interpretation of potential drug effects • Relevant renal parameters not to exceed ULN: creatinine, estimated GFR according to suitable formulae • Amylase and lipase to be interpreted in clinical context • Protocol to present rationale whether additional laboratory parameters required not to exceed reference ranges
Trials with ‘clinically established IMPs’ (e.g. bioequivalence trials for generic medicines applications**)**	• Slight elevation acceptable for hepatic parameters if no indication of apparent disease: 10% above ULN for ALT, 20% above ULN for AST or bilirubin (except in case of Gilbert’s disease*) *In case of Gilbert’s disease, see above • Slight elevation (10%) acceptable for renal parameters (except for creatinine**) if no indication of apparent disease **As creatinine especially in healthy male subjects also may reflect physical activity, protein intake by food, body height and muscle mass, some authors deem a slight elevation of creatinine up to 0.1 mg/dL above ULN as acceptable • Protocol to present rationale why these abnormal laboratory parameters seem acceptable

*IMP* investigational medicinal product, *ALT* alanine aminotransferase, *AST* aspartate aminotransferase, *AP* alkaline phosphatase, *GGT* gamma-glutamyltransferase, *ULN* upper limit of normal, *GFR* glomerular filtration rate

To rule out hypo-/hyperthyroidism (see also normal ranges for heart rate/pulse rate), it is recommended to determine thyroid stimulating hormone (TSH) levels.

#### ‘Trials with clinically established IMPs’

A slight (see Table 3) elevation above ULN is considered uncritical for hepatic integrity and renal function parameters (except for creatinine) as long as there are no other signs of underlying organic disease. The trial protocol should provide a rationale on further trial-specific parameters that need to be determined at screening (e.g. blood count, electrolytes, TSH) and of the ranges deemed acceptable; a risk-adapted approach is advised. Safety laboratory parameters out of normal ranges at screening might conceal potential adverse drug effects during treatment with the IMP leading to a decrease in the ‘signal to noise ratio’.

### Stopping rules

#### General aspects

A clear discontinuation strategy is an important safety aspect as outlined in the ICH-GCP guideline [6] and particularly applies to a phase I clinical trial [7]. Stopping rules for the whole trial, a single cohort and an individual subject have to be outlined in every trial protocol. Based on the risk evaluation of the IMP and the nature of the trial as described in the ‘*Guideline on strategies to identify and mitigate risks for first-in-human clinical trials with investigational medicinal products*’ [8], a dosing and discontinuation strategy must be established to control risk, e.g. by split-up of groups or staggered dosing.

The algorithm for decision making in dose escalation in healthy subjects published by Sibille et al. [9] is regarded as a reasonable approach for FIH trials, if not used as a fixed rule but as a recommendation to design criteria appropriate for a specific trial (see Table 4). With regard to the assessment of relatedness of serious adverse events (SAEs), a conservative approach is strongly recommended. Particularly in FIH trials, the information on human safety targets is very limited and solely derived from nonclinical data. Therefore, SAEs (e.g. myocardial infarction or stroke) in healthy volunteers should be handled as if they were IMP-related as long as other causes are not clearly established.

**Table 4:** Stopping rules for first-in-human trials

Individual subject	**•**Coding adverse events and laboratory abnormalities according to e.g. CTCAE criteria/grades may facilitate definition of stopping rules, even though CTCAE not really suitable for healthy subjects • 1 adverse event of severe intensity (Grade 3*) *Some stakeholders apply this stopping rule only in case of a causal relationship with the IMP • 1 serious adverse event • Relevant signs or symptoms affecting subject safety • Decision always taken by the investigator
Dose group/cohort (stop of further dose escalation)	•≥50% of subjects of the preceding dose step experienced adverse events of moderate (Grade 2, safety alert, ‘warning signal?’) or severe (Grade 3) intensity considered to be drug-related (selective unblinding) • 1 serious adverse event suspected to be drug-related (unblinding advised) = 1 serious adverse drug reaction • In case trial to be continued following safety consultation between all stakeholders, substantial amendment required
Termination of entire trial	• Decision taken by mutual agreement between investigator and sponsor

*IMP* investigational medicinal product, *CTCAE* Common Terminology Criteria for grading of Adverse Events developed by the National Cancer Institute (1 = mild, 2 = moderate, 3 = severe, 4 = life-threatening, 5 = death), version current at the time of publication of this consensus paper: http://evs.nci.nih.gov/ftp1/CTCAE/CTCAE_4.03_2010-06-14_QuickReference_5x7.pdf

An interim analysis of e.g. pharmacokinetic (PK) data (exposure versus no-adverse-effect-level, NOAEL) or safety data should be considered to enable a sound decision on the further conduct of the trial (e.g. further dose escalation), and the rationale for the decision whether PK data need to be available prior to the further conduct should be described in the trial protocol. A substantial amendment may be necessary to reflect potential changes in the subsequent conduct of the trial, depending on the procedures already described in the trial protocol.

For ‘*trials with clinically established IMPs*’, stopping rules follow the same principles even if sufficient information on the tolerable dose and the pertinent safety of the substance is available. Doses above clinically established ranges might increase the risk (see Table 5).

**Table 5:** Stopping rules for other/later phase I trials

• General risk assessment based on
- exposure (e. g. high exposure in drug-drug interaction trials, supra-therapeutic exposure in thorough QT trials)
- frequency of relevant adverse events
• Protocol to define stopping rules and decision making process for individual subjects, cohorts, and entire trial

### Consequences of the consensus

Based on 2 years’ experience with the consensus described in this paper, the German Competent Authority concluded that relevant protocol deficiencies regarding heart rate inclusion criteria showed a clear downtrend. There were no noteworthy deficiencies in safety laboratory inclusion criteria. German sponsors confirmed a high degree of acceptance of clinical trial applications when adopting the consensus criteria. Deficiency letters as well as trial amendments could be reduced.

## Discussion

To overcome deficiencies in clinical trial protocols identified by the German competent authority regarding key eligibility criteria and stopping rules for trials in healthy subjects, a discussion forum was hosted by the German not-for-profit Association for Applied Human Pharmacology (AGAH e.V.) and a consensus was reached between stakeholders from pharmaceutical industry, contract research organisations, academia, ethics committees and competent authority.

Normal ranges on its own cannot reliably distinguish a healthy from an unhealthy person or vice versa—unless they itself are used to define a disease—as they per definition only represent 95% of a reference population whereas 5% fall outside of this range. This is best characterised for laboratory values but also applies to other diagnostic findings. Systematic evaluations on the distribution of safety parameters in healthy individuals and their variations in respect with upper and lower limits of ‘normal’ are sparse and only empirical phase I trial data have been published. Moreover, it is well described that placebo and nocebo effects can influence clinical trial results [10, 11]. The following key safety data were addressed during the consensus debate.

### Distribution of cardiovascular safety parameters in healthy volunteers

Minor abnormalities in healthy subjects occur frequently. Sinus bradycardia as well as rhythm and conduction abnormalities have been described by several authors.

For instance, Hingorani et al. investigated ECGs in healthy subjects participating in phase I trials and assessed (1) the frequency of morphological abnormalities in 12-lead baseline ECGs [12] and (2) whether baseline abnormalities in 12-lead ECGs disappeared during the further course of a trial or new abnormalities occurred during administration of placebo [13].

Baseline 12-lead ECGs taken in 2458 healthy volunteers (aged 21 to 45 years) revealed rhythm abnormalities in 12.9% of subjects and conduction abnormalities in 5.7%. Specifically, sinus bradycardia was observed in 9.3% of subjects and first-degree AV block occurred in 2.2% of subjects of this age group [12].

Analyses of 16,472 ECGs from 420 healthy subjects (aged 18–76 years) demonstrated that transient ECG changes occur spontaneously on placebo exposure. About 43% of subjects with normal baseline ECGs (276/420 subjects; 65.7%) newly developed ECG abnormalities over the next 6 weeks. About 45% of subjects with abnormal 12-lead ECGs at baseline (144/420 subjects; 34.3%) spontaneously reverted to normal throughout the further course of the trial. Sinus bradycardia was present at baseline in 8.1% of subjects, was newly diagnosed on placebo in 13.6% of subjects and persisted throughout the trial in 2.1% of subjects. Sinus bradycardia is not uncommon in healthy subjects and may possibly be due to physiological changes in vagal tone, diurnal variations or the effect of food intake [13].

During 24-h ambulatory ECG recordings, Stinson et al. observed normal sinus rhythm in only 13% of 156 healthy volunteers throughout the entire observation period [14]*.* In one striking example from a case report, an idiosyncratic ventricular rhythm was found under placebo medication during a double-blind phase I study [15]*.*

### Distribution of safety laboratory values in healthy volunteers

Similar to ECG parameters, laboratory parameters deviating from the defined normal range have been described in healthy volunteers. Wensing and co-workers analysed the laboratory values of more than 17,000 blood samples taken from 3082 healthy volunteers (aged 18 to 55 years and in documented good health). Increased hepatic parameters were observed in a considerable number of subjects at screening (7.6% of subjects had ALT > ULN, 1.9% had AST > ULN, 12.3% had bilirubin >ULN). Likewise, following exposure to placebo (401 subjects), 13.7% of subjects had ALT > ULN, 3.2% had AST > ULN and 9.7% had bilirubin >ULN [16]. Elevated bilirubin values have also been demonstrated in approximately 10% of healthy volunteers recruited by a French phase I unit [17]. Rosenzweig et al. described elevation of aminotransferases in 20% of healthy subjects who received placebo [18]. Cai et al. showed that ALT levels during placebo administration were significantly higher than baseline levels in 481 healthy volunteers [19]*.* Microscopic haematuria is found in 9–18% of normal individuals [20]. Nutrition, in particular intake of meat, muscle mass and exercise were shown to significantly increase serum creatinine concentrations [21]. Those laboratory findings in obviously healthy subjects can be caused by genetic polymorphisms [22], by intraindividual short-term variations, within 1 day [23] and during 1 week, and long-term variations [24, 25] without any relevant underlying pathophysiological mechanisms being identified or being relevant for the inclusion in a phase I clinical trial. In 1997, Sibille et al. tried to introduce a more predictive definition of relevant laboratory alterations in general as the combination of the normal range limits and the spontaneous variations of parameters in healthy volunteers in their Phase I Unit [26]. This approach, however, has not been moved forward and has never been validated.

Taken together, these observations suggest that changes compared to screening/baseline parameters have to be interpreted carefully as they do not necessarily reflect adverse reactions induced by an IMP but may be due to spontaneous variations, placebo effects or may reflect the trial conditions [13, 18, 19, 27]. Thus, the assessment of safety signals emerging in a clinical trial can never be a simple tick box approach but always remains a complex medical decision which requires the clinical judgement of an adequately trained and experienced physician [7].

The considerations concerning the stopping rules for a single individual, a cohort or the entire trial are in line with the algorithm published by the French Club Phase I [9]. The grading system suggested by Sibille et al. is a reasonable approach to improve assessment of adverse events [9] as existing grading systems (e.g. CTCAE) [28] have not been developed with the focus on phase I trials and thus may have relevant limitations if used in this context.

Defining acceptable normal ranges for key safety parameters, timing and frequency of safety assessments as well as adequate stopping criteria seems particularly important in context of the current debate about the safety of healthy subjects in phase I trials. A healthy subject died in the multiple ascending dose part of a FIH trial after having been exposed to a newly developed FAAH inhibitor [1, 29]. Following the fifth administration, one of six subjects on active treatment with the 50 mg dose developed a serious condition and was hospitalised. The investigator did not initially consider the relationship between the acute symptoms presented by the subject and the molecule tested to be possible. The other five volunteers received their sixth dose the next morning without waiting for the results of the ongoing tests on the hospitalised volunteer. The other five volunteers receiving active treatment were in turn hospitalised after their sixth dose but not the two volunteers on placebo. Thereafter, the trial was suspended.

Adequate stopping rules could not have prevented the observed serious condition in one healthy subject in this FIH trial but may have helped in preventing further dosing of other subjects in the same dose group who were hospitalised subsequently.

## Conclusion

A consensus was achieved among stakeholders on a standardised approach for the evaluation of healthy subjects before enrolment in phase I trials and during dosing.

The consensus shall help sponsors, investigators and clinical trial staff to further improve subject safety and facilitate interaction with regulatory bodies concerning key safety issues in phase I trials.
